# Assessment of Maternal Macular Pigment Optical Density (MPOD) as a Potential Marker for Dietary Carotenoid Intake during Lactation in Humans

**DOI:** 10.3390/nu14010182

**Published:** 2021-12-31

**Authors:** Ateka Al-Hassan, Rutvi Vyas, Yue Zhang, Michaela Sisitsky, Borjan Gagoski, Jonathan S. Litt, Ryan J. Larsen, Matthew J. Kuchan, John B. Lasekan, Brad P. Sutton, Patricia Ellen Grant, Yangming Ou, Sarah U. Morton

**Affiliations:** 1 Division of Newborn Medicine, Boston Children’s Hospital, Boston, MA 02115, USA; atekaalhassan@gmail.com (A.A.-H.); rutvi.vyas@childrens.harvard.edu (R.V.); ellen.grant@childrens.harvard.edu (P.E.G.); 2 Fetal-Neonatal Neuroimaging and Developmental Science Center, Boston Children’s Hospital, Boston, MA 02115, USA; zhangyue11022@whu.edu.cn (Y.Z.); michaela.sisitsky@childrens.harvard.edu (M.S.); Borjan.gagoski@childrens.harvard.edu (B.G.); 3 Department of Radiology, Boston Children’s Hospital, Boston, MA 02115, USA; 4 Department of Neonatology, Beth Israel Deaconess Medical Center, Boston, MA 02115, USA; jlitt@bidmc.harvard.edu; 5 Beckman Institute, University of Illinois at Urbana-Champaign, Urbana, IL 61801, USA; larsen@illinois.edu (R.J.L.); bsutton@illinois.edu (B.P.S.); 6 Nutrition and Brain Health, Clinical Research, Abbott Nutrition, Columbus, OH 43219, USA; matthew.kuchan@abbott.com (M.J.K.); john.lasekan@abbott.com (J.B.L.); 7 Department of Bioengineering, University of Illinois at Urbana-Champaign, Urbana, IL 61801, USA; 8 Department of Pediatrics, Harvard Medical School, Boston, MA 02115, USA

**Keywords:** macular pigment optical density (MPOD), nutrition, postpartum, carotenoids, breastmilk, lactation

## Abstract

Pregnancy and lactation can change the maternal nutrient reserve. Non-invasive, quantitative markers of maternal nutrient intake could enable personalized dietary recommendations that improve health outcomes in mothers and infants. Macular pigment optical density (MPOD) is a candidate marker, as MPOD values generally reflect carotenoid intake. We evaluated the association of MPOD with dietary and breastmilk carotenoids in postpartum women. MPOD measurements and dietary intake of five carotenoids were obtained from 80 mothers in the first three months postpartum. Breastmilk samples from a subset of mothers were analyzed to determine their nutrient composition. The association between MPOD and dietary or breastmilk carotenoids was quantitatively assessed to better understand the availability and mobilization of carotenoids. Our results showed that dietary α-carotene was positively correlated with MPOD. Of the breastmilk carotenoids, 13-cis-lutein and trans-lutein were correlated with MPOD when controlled for the total lutein in breastmilk. Other carotenoids in breastmilk were not associated with MPOD. Maternal MPOD is positively correlated with dietary intake of α-carotene in the early postpartum period, as well as with the breastmilk content of lutein. MPOD may serve as a potential marker for the intake of carotenoids, especially α-carotene, in mothers in the early postpartum period.

## 1. Introduction

Breastmilk is the recommended diet for infants, and the maternal nutrient status during lactation can affect the nutrient content of breastmilk [1,2]. As nutrient intake during this critical period of brain development can impact neurodevelopmental outcomes, identifying modifiable factors that optimize the nutrient content of breastmilk could improve health outcomes for infants [3,4,5,6,7,8]. Carotenoids function as antioxidants, and three of them (β-carotene, lycopene, and β-cryptoxanthin) can serve as precursors of vitamin A and influence retinoid signaling levels [9]. They have been shown to play a role in early visual and cognitive development and are primarily obtained from the mother during pregnancy or later from breastmilk [10]. Dietary lutein intake correlates with lutein concentrations in breastmilk [11], and maternal supplementation with lutein can increase the amount in breastmilk [12]. However, the individualized assessment of maternal nutrition remains a challenge. Non-invasive quantitative markers for maternal intake of nutrients such as carotenoids would be useful to identify dietary factors that affect maternal nutrient status during pregnancy and lactation, and in the future could provide an opportunity for individualized nutritional recommendations.

Postpartum intake of carotenoids can be highly variable between mothers [13]. The food frequency questionnaire (FFQ) is a widely used assessment tool for nutrient intake. FFQs survey the frequency and amount of consumed food items [14]. An FFQ is a non-invasive quantitative measurement, but it requires specialized analysis to convert food data to nutrient intake values. Another tool, macular pigment optical density (MPOD), uses a tabletop instrument to quantify macular pigment in the retina. Macular pigment is composed of lutein, zeaxanthin, and meso-zeaxanthin. While meso-zeaxanthin is believed to be generated enzymatically from lutein in the retina, lutein and zeaxanthin cannot be synthesized by humans and therefore must be obtained from the diet [15,16,17]. For this reason, MPOD has been used to assess the status of carotenoids, such as lutein and zeaxanthin, in the general population [18,19,20,21,22]. Similar to FFQs, MPOD can be measured non-invasively; however, the measurement takes less than 5 minutes and generates a single objective value. In addition, MPOD reflects the absorption and utilization of carotenoids and therefore could reflect nutritional status similar to FFQs. Finally, as carotenoids are widely distributed in foods such as fruits and vegetables, measuring carotenoids could provide a useful insight into the intake of these healthy foods.

These features motivated us to evaluate whether MPOD could represent FFQ-derived maternal carotenoid intake. Specifically, we measured food intake and MPOD in the first 3 months postpartum from 80 mothers and collected breastmilk samples from a subset of mothers. The main objective of this cross-sectional study is to determine if MPOD is associated with dietary intake of carotenoids in lactating mothers. Secondly, we assessed whether the MPOD measurement was indicative of the mother’s carotenoid delivery to her infant via breastmilk.

## 2. Materials and Methods

### 2.1. Study Population

Data were obtained during a prospective observational study investigating the impact of nutrition on infant brain development (clinicaltrials.gov registration #NCT02058225, accessed on 24 October 2018). The research protocol was approved by the Institutional Review Boards at Boston Children’s Hospital, Brigham and Women’s Hospital, and Beth Israel Deaconess Medical Center. A total of 161 mother–infant dyads were recruited to the trial from 2013 to 2016. Written informed consent was obtained from each participant at the bedside after delivery. Inclusion criteria included: (a) mothers who delivered full-term infants at Brigham and Women’s or Beth Israel; (b) mothers with no known medical problems or pregnancy complications. Eighty mothers with both MPOD and FFQ data available were included in this study. Demographic information including age, race, ethnic groups, and education levels were obtained through questionnaires and are described in Table 1.

### 2.2. Study Measurements

#### 2.2.1. Macular Pigment Optical Density (MPOD) Measurement

MPOD was measured from 90 mothers in the early postpartum period (median (IQR) = 30 (22–59) days) using heterochromatic flicker photometry [23] (Supplemental Methods). The measurements were taken with the macular densitometer (Macular Metrics II, LLC, Rehoboth, MA, USA). To detect the flicker sensation (i.e., amount of light absorbed by the macular pigment), blue and green light were temporally modulated, based on a null adjustment procedure, where a good starting blue-to-green ratio for the individual participant was determined, and the participants were asked to confirm when a flicker was perceived. This flicker detection was measured in the right eye both at the center of the fovea and at the perifovea (7 degrees from the fovea). By comparing the foveal and parafoveal measurements, the densitometer generated the MPOD values, thus giving an estimation of amount of macular pigment in a participant. MPOD values typically range from 0 to 1 and are linearly related to the amount of macular pigment [18,24]. Measurements with values < 0.1 (*n* = 7) or if obtained from the left eye (*n* = 3) were excluded from the analysis for accuracy and consistency. MPOD measurements from 80 mothers (mean ± standard deviation (SD) = 0.38 ± 0.18) were included in the final analysis.

#### 2.2.2. Maternal Dietary Intake 

An FFQ (Block 2005 FFQ) administered by NutritionQuest was completed by each participant in the early postpartum period (median (IQR) = 35 (23–60) days) to assess their dietary intake over the past three months (nutritionquest.com/assessment/list-of-questionnaires-and-screeners, accessed on 24 October 2018). The questionnaire included 139 questions, which were converted to the average daily intake of 73 nutrients. Of these, 5 nutrients in the vitamins and antioxidants category were carotenoids: α-carotene, β-carotene, lycopene, β-cryptoxanthin, and combined lutein-zeaxanthin. The others were non-carotenoids and not included in this analysis. Body mass index (BMI), defined as body weight (kilogram) divided by height (meter) squared, was also collected as part of FFQ. All FFQs were collected within 2 (1–6) days (median (IQR)) of the MPOD measurements.

#### 2.2.3. Breastmilk

Mothers were asked to collect 50–100 mL of breastmilk using a breast pump at least 2 h after the last milk expression, excluding the first morning milk. Milk was frozen and brought to the research visit. The breastmilk samples were collected approximately 3 months postpartum (median (IQR) = 108 (99–119) days). The milk samples were then stored at −80 °C and were shipped to Abbott Inc. for analysis. The carotenoid concentrations in the breastmilk were obtained (Supplemental Table S1) using the analysis procedures as described by Xu et al. [25]. In brief, milk samples were mixed with water and tetrahydrafuran, then saponified for 30 min at room temperature with 5% methanolic potassium hydroxide. Target analytes were extracted using butylated hydroxytoluene in a mixture of dichloromethane, petro ether, and hexane. Extracts were dried, reconstituted in ethanol, and run on an YMC C30 column at a temperature of 23 °C using an Agilent 1290 Infinity high-performance liquid chromatography system. The linear assay range was 10–250 μg/L with a linear coefficient value of >0.9990 for both lutein and zeaxanthin. Of the 80 mothers who were included in the study, 38 mothers had all three research measurements: MPOD, dietary intake, and breastmilk samples.

### 2.3. Statistical Analysis

Associations between MPOD measurements and maternal intake of each of the 5 carotenoids in the postpartum period were calculated in SAS Studio (version 3.8, SAS Institute Inc., Cary, NC, USA) using a linear regression model. Of the 80 participants, 12 participants had both MPOD measurements and dietary intake information obtained at 2 timepoints in the early postpartum period, in which case average values were used. The significance of association was computed with and without considering maternal age, BMI, and number of days postpartum (daysPP) at the time of measurement as covariates to quantify whether these maternal factors mediate the associations. Additionally, a correlation matrix of the dietary carotenoids was calculated to characterize their degree of independence. Benjamini–Hochberg (BH) method was used to correct for multiple comparisons, and the level of significance was set to be q < 0.05. 

Furthermore, the association between the MPOD measurements and the carotenoids in breastmilk was calculated using a linear regression model, both with and without adjusting for maternal age, BMI, and number of days postpartum (daysPP) at the time of breastmilk collection as covariates. For the carotenoids, lutein and β-carotene, where both cis and trans-isomers were measured, the association between the sub-components and MPOD was adjusted for the total concentration of the carotenoids to account for the complementary effect of the isomers. Correction of multiple comparisons was carried out using BH method with 0.05 level of significance.

## 3. Results

### 3.1. Cohort Description

Of the 161 mother–infant dyads originally recruited, 80 had MPOD values that passed quality control filtering (Supplemental Figure S1). Thirty-eight mothers with MPOD values also provided a breastmilk sample. Comparing the intake of carotenoids between mothers who did and did not provide a breastmilk sample, there was no difference in carotenoid intake (Supplemental Table S2).

### 3.2. Serial MPOD Measures

Twenty-one mothers had MPOD values measured at both 1 and 3 months postpartum. MPOD values were lower at 3 months (average 0.3755 vs. 0.3175, Wilcoxon signed-rank test *p*-value 0.0075; Supplemental Figure S2).

### 3.3. MPOD-Carotenoid Association

**Figure 1:**
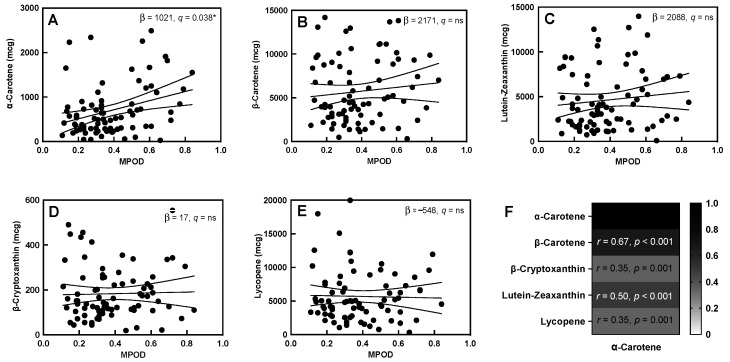
MPOD and dietary carotenoids. (**A**–**E**) Association between MPOD and all five dietary carotenoids (slope of the linear association and q-values are displayed in each box). (**F**) Correlation of dietary α-carotene with other dietary carotenoids (correlation coefficients and p-values are displayed in each cell). “ns” in the figure represents “not significant”. “*” indicates a significant correlation with q < 0.05, Solid lines indicate the slope of the association, with 95% confidence intervals indicated with dashed lines.

Dietary α-carotene had a positive association with MPOD (R^2^ = 0.10, q = 0.04, Figure 1A, Supplemental Table S3) in the unadjusted comparison, but it was only nominally associated when adjusted for maternal age, BMI, and daysPP at the time of measurement (R^2^ = 0.17, q = 0.05). The other dietary carotenoids, β-carotene, lutein-zeaxanthin, β-cryptoxanthin, and lycopene, did not show an association with maternal MPOD (Figure 1B–E) either in the unadjusted or the adjusted model. Dietary α-carotene was positively associated with the intake of each of the other carotenoids, especially β-carotene and lutein-zeaxanthin (Figure 1F). Dietary β-carotene and lutein-zeaxanthin were also positively correlated (Supplemental Figure S3).

**Figure 2:**
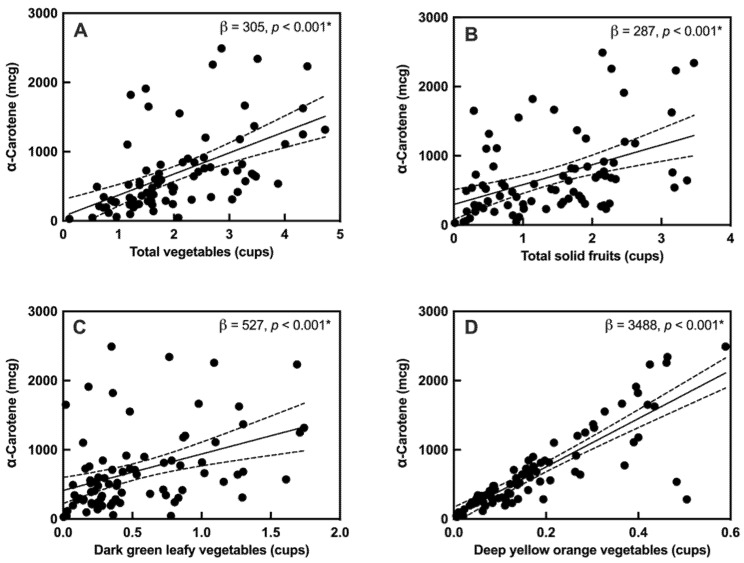
Associations between α-carotene and carotenoid-rich foods: (**A**) total vegetables, (**B**) total fruits (solid), (**C**) dark green leafy vegetables, and (**D**) deep yellow, orange-colored vegetables. “*” indicates a significant correlation with q < 0.05, Solid lines indicate the slope of the association, with 95% confidence intervals indicated with dashed lines.

Given the association between MPOD and α-carotene, the intake of carotenoid-rich foods such as fruits and vegetables, obtained from the FFQs, were assessed for association with dietary α-carotene using a linear regression model. The overall intake of vegetables, particularly dark green leafy and deep yellow, orange-colored vegetables, and the intake of solid fruits showed a strong positive relation with α-carotene (Figure 2). However, MPOD was not directly correlated with the intake of vegetables (Supplemental Table S4).

### 3.4. Material Diet-Breastmilk Association 

The maternal intake of carotenoids was not correlated with breastmilk carotenoid contents, in either an unadjusted model or when accounting for maternal age, BMI, and days postpartum (Supplemental Table S5).

### 3.5. MPOD-Breastmilk Association 

**Figure 3:**
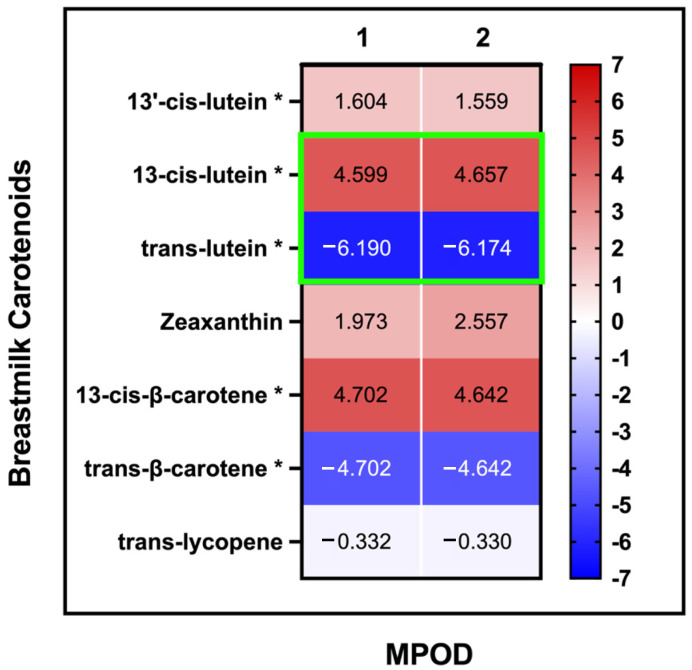
MPOD and breastmilk carotenoids: association of MPOD measurements with carotenoids in breastmilk: (**1**) no covariates (**2**) with maternal age, BMI, and daysPP as covariates (the slope (β value) for each linear association is displayed in each cell). Significant assocation values are highlighted with green outline for 13-cis-lutein and trans-lutein. * For these variables, the total concentration of the carotenoids was included as covariates.

The fraction of 13-cis-lutein in breastmilk was nominally positively associated with MPOD (R^2^ = 0.82, q = 0.04, Figure 3, Supplemental Table S6), while the fraction of trans-lutein in breastmilk was negatively associated with MPOD (R^2^ = −0.96, q = 0.04) when accounted for the total lutein in breastmilk. This association remained nominal even when maternal age, maternal BMI, and daysPP were used as covariates (R^2^ = −0.83, q = 0.05; R^2^ = −0.96, q = 0.04). None of the other breastmilk carotenoids showed any association with the MPOD measurements.

## 4. Discussion

This study aimed to identify whether maternal MPOD reflects maternal dietary carotenoid intake and carotenoid concentrations in breastmilk during the postpartum period, which has rarely been studied. Clinical documentation of MPOD as a quantitative, non-invasive marker for maternal dietary carotenoids and levels in breastmilk could help establish recommendations for carotenoids, which are important for health and neurodevelopmental outcomes in infants [4,6,10,26,27,28,29]. Significant associations between MPOD and carotenoid intake, especially lutein and zeaxanthin, have been previously found in general adult populations [16,22,30,31,32,33,34]. Most of these studies have not investigated the association of MPOD with other dietary carotenoids such as α-carotene, β-carotene, or lycopene. Surprisingly, the current data indicate that dietary intake of the macular carotenoids was not correlated with MPOD in the postpartum period. Moreover, in contrast to previous studies that demonstrated an increase in breastmilk lutein content after maternal dietary supplementation, we found no association with dietary intake of lutein and breastmilk lutein contents. Instead, our results reveal a significant association between MPOD and α-carotene intake, as well as the breastmilk contents of lutein isoforms.

### 4.1. MPOD and α-Carotene Intake: Marker of Healthy Diet?

MPOD was most strongly correlated with a maternal intake of α-carotene, despite α-carotene not being a macular pigment. In our cohort, α-carotene was correlated with the intake of dark green leafy and deep yellow, orange-colored vegetables and solid fruits, and therefore may have been a proxy marker for a healthy diet rich in carotenoid-dense foods. Therefore, this leads us to consider whether MPOD might serve as a useful indicator of the intake of non-macular carotenoids and/or healthy vegetables and fruits in women postpartum. However, MPOD was not directly correlated with the intake of carotenoid-rich vegetables, nor with the intake of non-macular carotenoids. Instead, the correlation of MPOD with dietary α-carotene may be due to secondary effects of α-carotene on macular health during the postpartum period or due to difficulties in accurately ascertaining intake of lutein-zeaxanthin from FFQ. This could be due to a small sample size, confounders in the measurement of macular carotenoids, or an unreliable FFQ assessment of macular carotenoids. The strong association of dietary α-carotene with dietary lutein-zeaxanthin and β-carotene suggests associations with α-carotene may also be true for lutein-zeaxanthin if replicated in a larger cohort. Together, these results suggest that maternal MPOD warrants further study as a marker of carotenoid status in the early postpartum phase.

### 4.2. Lutein Isoforms

Though MPOD is well known to be correlated with lutein-zeaxanthin intake, the relationship of MPOD with breastmilk carotenoids has not been previously determined. We hypothesized that MPOD would be positively correlated with lutein concentrations in breastmilk [11], as those have also been shown to be positively correlated with dietary lutein intake [12]. Interestingly, breastmilk cis and trans-lutein were positively and negatively correlated with MPOD, respectively. This could be due to differences in the foods containing the two isoforms, differences in isoform bioavailability, or differences in lutein metabolism and storage during lactation. Cis-lutein is generated from naturally occurring trans-lutein during the processing and/or cooking of vegetables, fruits, and eggs [35]. Thus, higher breastmilk cis-lutein likely reflects a higher maternal intake of cooked and/or processed foods that contained trans-lutein in their pre-processed forms. On the other hand, trans-lutein is the naturally occurring isomer, and the infant brain is strongly selective for trans-lutein as compared to cis-lutein, displaying a higher trans-lutein-to-cis-lutein ratio [36]. This suggests that trans-lutein might be more concentrated in human tissues. Mobilization of trans-lutein from the macular carotenoids, or preferential transport of trans-lutein into breastmilk from dietary intake or adipose tissue mobilization, could explain the negative correlation of MPOD with breastmilk trans-lutein concentrations.

Limitations of our current study include the use of a single MPOD measurement and small sample size, though this is similar to other MPOD studies in other life stages. Small sample sizes both limit power to detect true correlations and can increase the likelihood of detecting an incorrect correlation due to bias within the cohort. In addition, MPOD was measured only in the postpartum period, but not in other periods. Future studies are needed to determine how diet affects MPOD during pregnancy [4,7,10,27,28,37]. Our methods were unable to separate dietary lutein and zeaxanthin due to the configurations in the FFQ analysis. Moreover, α-carotene and cis-lycopene in breastmilk could not be measured accurately and thus could not be included in the analysis. Including measurements of plasma concentrations of carotenoids in women pre and postpartum may also be beneficial. Finally, a multivariate approach looking at other nutrients from the maternal dietary intake and their effects on MPOD and changes in breastmilk contents would be important to include in such studies.

Despite these limitations, this is the first quantitative study of maternal MPOD and nutritional correlates in the postpartum period for a term-born population. In summary, MPOD measurements could reflect the intake of carotenoids, particularly α-carotene, in the early postpartum period. The lack of direct association between maternal dietary lutein-zeaxanthin and MPOD may reflect additional complexities in the regulation of macular pigment deposition during lactation. The association of a lutein component in breastmilk with MPOD may suggest selective transport of lutein-zeaxanthin to breastmilk in mothers. Future studies to determine the utility of prenatal MPOD measurements would be an important next step in determining the utility of MPOD as a non-invasive indicator of maternal nutrient status in the postpartum period.

## Figures and Tables

**Table 1:** Demographics of participants (*N* = 80).

Maternal Characteristics, median (inter-quartile range, IQR)	
Age (years)	33 (30–36)
Body mass index (BMI) (kg/m^2^)	27 (25–30)
Time of MPOD measurements postpartum (days)	30 (22–59)
Time of FFQ collection postpartum (days)	35 (23–60)
Time of breastmilk sample collection (days)	108 (99–119)
Ethnicity, number (%)	
Hispanic or Latino	6 (7.5%)
Not Hispanic or Latino	65 (87.5%)
Not reported	4 (5.0%)
Race, number (%)	
White	56 (70.0%)
Black or African American	11 (13.8%)
Asian	6 (7.5%)
More than one race	2 (2.5%)
Not reported	5 (6.2%)
Highest Degree Earned, number (%)	
High school diploma or equivalency (GED)	7 (8.7%)
Associate degree (junior college)	7 (8.7%)
Bachelor’s degree	31 (38.7%)
Master’s degree	21 (26.3%)
Doctorate professional	11 (13.8%)
Other	3 (3.8%)

MPOD: macular pigment optical density; FFQ: food frequency questionnaire; GED: general educational development.

## Data Availability

The data presented in this study are available on request from the corresponding author. The data are not publicly available due to lack of consent.

## Supplementary Materials

The following supporting information can be downloaded at: https://www.mdpi.com/article/10.3390/nu14010182/s1, Figure S1: Correlation matrix of dietary carotenoids (correlation coefficients are displayed in each box); Figure S2: Serial MPOD measures; Figure S3: Correlation matrix of dietary carotenoids (correlation coefficients are displayed in each box); Table S1: Unadjusted and adjusted linear model of the association between MPOD and dietary carotenoids; Table S2: Lack of correlation between MPOD and carotenoid-rich foods; Table S3: Unadjusted and adjusted linear model of the association between MPOD and breastmilk carotenoids. Significant association values are highlighted with green outline; Table S4: Lack of correlation between MPOD and carotenoid-rich foods; Table S5: Correlation of maternal carotenoid intake with breastmilk contents; Table S6: Unadjusted and adjusted linear model of the association between MPOD and breastmilk carotenoids. Significant association values are highlighted with green outline; Methods: densitometer operating instructions.

## References

[1] Ballard O., Morrow A.L. (2013). Human milk composition: Nutrients and bioactive factors. Pediatr. Clin. N. Am..

[2] Bravi F., Wiens F., Decarli A., Pont A.D., Agostoni C., Ferraroni M. (2016). Impact of maternal nutrition on breast-milk composition: A systematic review. Am. J. Clin. Nutr..

[3] Chen X., Zhao D., Mao X., Xia Y., Baker P.N., Zhang H. (2016). Maternal Dietary Patterns and Pregnancy Outcome. Nutrients.

[4] Dani C., Lori I., Favelli F., Frosini S., Messner H., Wanker P., De Marini S., Oretti C., Boldrini A., Massimiliano C. (2012). Lutein and zeaxanthin supplementation in preterm infants to prevent retinopathy of prema-turity: A randomized controlled study. J. Matern. Fetal. Neonatal. Med..

[5] Koletzko B., Godfrey K.M., Poston L., Szajewska H., Van Goudoever J.B., De Waard M., Brands B., Grivell R.M., Deussen A.R., Dodd J.M. (2019). Nutrition during pregnancy, lactation and early childhood and its implications for maternal and long-term child health: The early nutrition project recommendations. Ann. Nutr. Metab..

[6] Mulder K.A., Innis S.M., Rasmussen B.F., Wu B.T., Richardson K.J., Hasman D. (2014). Plasma lutein concentrations are related to dietary intake, but unrelated to dietary saturated fat or cognition in young children. J. Nutr. Sci..

[7] Sasano H., Obana A., Sharifzadeh M., Bernstein P.S., Okazaki S., Gohto Y., Seto T., Gellermann W. (2018). Optical Detection of Macular Pigment Formation in Premature Infants. Transl. Vis. Sci. Technol..

[8] Vohr B.R., Poggi Davis E., Wanke C.A., Krebs N.F. (2017). Neurodevelopment: The Impact of Nutrition and Inflammation During Pre-conception and Pregnancy in Low-Resource Settings. Pediatrics.

[9] De Souza Mesquita L.M., Mennitti L.V., De Rosso V.V., Pisani L.P. (2021). The role of vitamin A and its pro-vitamin carotenoids in fetal and neonatal programming: Gaps in knowledge and metabolic pathways. Nutr. Rev..

[10] Zielińska M.A., Wesołowska A., Pawlus B., Hamułka J. (2017). Health Effects of Carotenoids during Pregnancy and Lactation. Nutrients.

[11] Kim H., Bae T.J., Jung B.M., Yi H., Jung J.A., Chang N. (2017). Association between lutein intake and lutein concentrations in human milk samples from lactating mothers in South Korea. Eur. J. Clin. Nutr..

[12] Sherry C.L., Oliver J.S., Renzi-Hammond L., Marriage B.J. (2014). Lutein Supplementation Increases Breast Milk and Plasma Lutein Concentrations in Lactating Women and Infant Plasma Concentrations but Does Not Affect Other Carotenoids. J. Nutr..

[13] Zielinska M.A., Hamulka J., Wesolowska A. (2019). Carotenoid Content in Breastmilk in the 3rd and 6th Month of Lactation and Its Associations with Maternal Dietary Intake and Anthropometric Characteristics. Nutrients.

[14] Steinemann N., Grize L., Ziesemer K., Kauf P., Probst-Hensch N., Brombach C. (2017). Relative validation of a food frequency questionnaire to estimate food intake in an adult population. Food Nutr. Res..

[15] Abdel-Aal E.S.M., Akhtar H., Zaheer K., Ali R. (2013). Dietary sources of lutein and zeaxanthin carotenoids and their role in eye health. Nutrients.

[16] Ma L., Liu R., Du J.H., Liu T., Wu S.S., Liu X.H. (2016). Lutein, Zeaxanthin and Meso-zeaxanthin Supplementation Associated with Macular Pigment Optical Density. Nutrients.

[17] Raman G., Haslam D., Avendano E., Johnson E.J. (2019). Lutein/zeaxanthin intake and visual outcomes in adults with healthy eyes: Qualitative gap analysis. Cogent Med..

[18] Bernstein P.S., Delori F.C., Richer S., Van Kuijk F.J., Wenzel A.J. (2010). The value of measurement of macular carotenoid pigment optical densities and distributions in age-related macular degeneration and other retinal disorders. Vis. Res..

[19] Curran-Celentano J., Hammond B.R., Ciulla T.A., Cooper D.A., Pratt L.M., Danis R.B. (2001). Relation between dietary intake, serum concentrations, and retinal concentrations of lutein and zeaxanthin in adults in a Midwest population. Am. J. Clin. Nutr..

[20] Meagher K.A., Thurnham D.I., Beatty S., Howard A.N., Connolly E., Cummins W., Nolan J.M. (2012). Serum response to supplemental macular carotenoids in subjects with and without age-related macular degeneration. Br. J. Nutr..

[21] Scott T., Rasmussen H.M., Chen C.-Y.O., Johnson E.J. (2017). Avocado consumption increases macular pigment density in older adults: A randomized, controlled trial. Nutrients.

[22] Wilson L.M., Tharmarajah S., Jia Y., Semba R.D., Schaumberg D.A., Robinson K.A. (2021). The Effect of Lutein/Zeaxanthin Intake on Human Macular Pigment Optical Density: A Systematic Review and Meta-Analysis. Adv. Nutr. Int. Rev. J..

[23] Bone R.A., Landrum J.T. (2004). Heterochromatic flicker photometry. Arch. Biochem. Biophys..

[24] Snodderly D., Handelman G.J., Adler A.J. (1991). Distribution of individual macular pigment carotenoids in central retina of macaque and squirrel monkeys. Investig. Ophthalmol. Vis. Sci..

[25] Xu X., Zhao X., Berde Y., Low Y.L., Kuchan M.J. (2018). Milk and Plasma Lutein and Zeaxanthin Concentrations in Chinese Breast-Feeding Mother–Infant Dyads with Healthy Maternal Fruit and Vegetable Intake. J. Am. Coll. Nutr..

[26] Renzi-Hammond L.M., Bovier E.R., Fletcher L.M., Miller L.S., Mewborn C.M., Lindbergh C.A., Baxter J.H., Hammond B.R. (2017). Effects of a Lutein and Zeaxanthin Intervention on Cognitive Function: A Randomized, Double-Masked, Placebo-Controlled Trial of Younger Healthy Adults. Nutrients.

[27] Manzoni P., Stolfi I., Pedicino R., Vagnarelli F., Mosca F., Pugni L., Bollani L., Pozzi M., Gomez K., Tzialla C. (2013). Human milk feeding prevents retinopathy of prematurity (ROP) in preterm VLBW neonates. Early Hum. Dev..

[28] Henriksen B.S., Chan G., Hoffman R.O., Sharifzadeh M., Ermakov I.V., Gellermann W., Bernstein P.S. (2013). Interrelationships between maternal carotenoid status and newborn infant macular pigment optical density and carotenoid status. Investig. Ophthalmol. Vis. Sci..

[29] Barnett S.M., Khan N.A., Walk A.M., Raine L.B., Moulton C., Cohen N.J., Kramer A.F., Hammond B.R., Renzi-Hammond L., Hillman C.H. (2017). Macular pigment optical density is positively associated with academic performance among preadolescent children. Nutr. Neurosci..

[30] Beatty S., Nolan J., Kavanagh H., Donovan O.O. (2004). Macular pigment optical density and its relationship with serum and dietary levels of lutein and zeaxanthin. Arch. Biochem. Biophys..

[31] Eggersdorfer M., Wyss A. (2018). Carotenoids in human nutrition and health. Arch. Biochem. Biophys..

[32] Hammond B.R., Ciulla T.A., Snodderly D. (2002). Macular pigment density is reduced in obese subjects. Investig. Ophthalmol. Vis. Sci..

[33] Hammond B., Johnson E.J., Russell R.M., Krinsky N.I., Yeum K.J., Edwards R.B., Snodderly D. (1997). Dietary modification of human macular pigment density. Investig. Ophthalmol. Vis. Sci..

[34] Nolan J.M., Stack J., O’Connell E., Beatty S. (2007). The relationships between macular pigment optical density and its constituent carotenoids in diet and serum. Investig. Ophthalmol. Vis. Sci..

[35] Ochoa Becerra M., Mojica Contreras L., Hsieh Lo M., Mateos Díaz J., Castillo Herrera G. (2020). Lutein as a functional food ingredient: Stability and bioavailability. J. Funct. Foods.

[36] Tanprasertsuk J., Li B., Bernstein P.S., Vishwanathan R., Johnson M.A., Poon L., Johnson E.J. (2016). Relationship between Concentrations of Lutein and StARD3 among Pediatric and Geriatric Human Brain Tissue. PLoS ONE.

[37] Osborne M., Porto K., Patel K., Soviravong S., White R., Barrow K., Luthra N., Hammond B.R., Stringham J., Tracy Q. Investigating macular pigment optical density in pregnant mothers during prenatal and postnatal stages. Proceedings of the 38th Annual Convention of the Behavioral Sciences, Psi Chi International Honor Society.

