# Supplementary material for: Assessment of Maternal Macular Pigment Optical Density (MPOD) as a Potential Marker for Dietary Carotenoid Intake during Lactation in Humans
Source: Nutrients. 2021 Dec 31;14(1):182. doi: 10.3390/nu14010182 (PMC8746783; doi:10.3390/nu14010182)
Supplement: Supplementary file 1 [file nutrients-14-00182-s001.zip › nutrients-1498424-Supplementary Methods.pdf]

Densitometer Operating Instructions

Start up instructions to the operator

Confirm presence of the provided flash drive in USB port before turning on the densitometer. **ATTENTION:** If you remove the flash drive from the USB port for data transfer, before you return the flash drive to the port you must turn off the densitometer. Reinsert the flash drive in the USB port and restart the densitometer to continue collecting patient data files.

1) Power on at least 10 minutes before running your first patient. You will be presented with the following Start Up screen.
2) Input patient information (e.g., name, age, gender, eye). Touch the desired field (e.g., Name) to access an onscreen keyboard for data entry. You will not be able to proceed if the Name field is not populated. If needed, adjust the date and time. Touching the date-time field in the upper right hand corner will produce a pop up window for adjusting those values.

The Start Up Screen

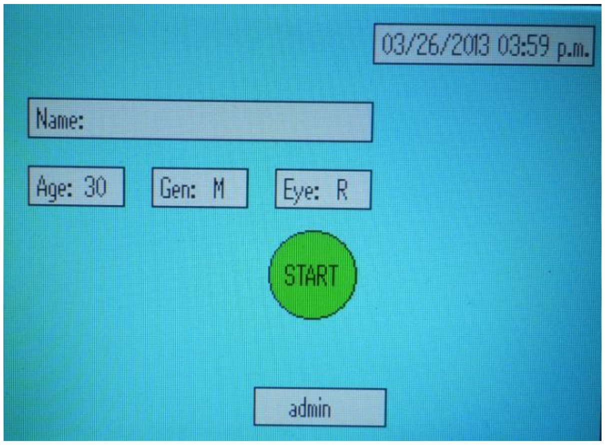

Operator instructions to patient for foveal (centrally-fixated) viewing

1) Use the provided foveal stimulus example to describe the task, including **careful fixation** of the central fixation point, initiation of a trial by pushing the patient response button, and responding to the onset of flicker by pushing the patient response button again.
2) Advise your patient that the audible triple beep after each response is their signal to initiate the next trial by pushing the response button. Again, at the onset of flicker they are to push the button to record their response. This procedure is repeated through completion of foveal viewing. Reminding the patient to fixate at each trial is desirable.

Running the session

1) Press the START button on the Start Up screen (above) to advance the program to the foveal (centrally-fixated) CFF mode (see CFF screen below). An audible triple-beep is heard indicating that the program is ready to start the first CFF trial.

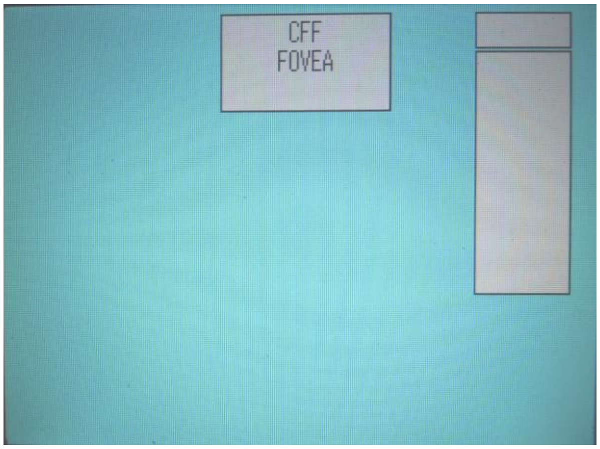

Foveal CFF (1 deg. target)

The patient is initially presented with a non-flickering centrally-fixated target. Have the patient confirm that the stimulus (central target & fixation point) is in good focus. If not, have them turn the adjustable eyepiece to bring the stimulus into focus. When ready the patient should press the response button to initiate the first trial and again as soon as flicker is perceived to record their response. After each response an audible triple-beep is sounded indicating the program is ready to proceed. Upon hearing the triple-beep the patient initiates the next trial by pushing the response button. This is repeated for a total of 5 CFF trials. The running average is displayed on the left of the central CFF FOVEA field. When CFF is completed the program eliminates the highest and lowest responses and displays the average of the remaining 3 responses on the right side of the CFF FOVEA field. This CFF average provides a starting alternation frequency, specific to the patient, for the foveal bracketing phase.

CFF Results Screen

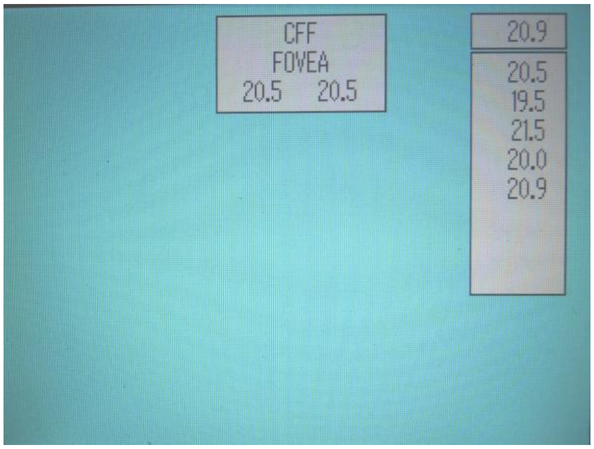

Foveal flicker nulling (1 deg. target)

When the last CFF trial is completed the stimulus is turned off for a few seconds while the program automatically advances to the next phase of the program. The operator is presented with the following screen and the patient is presented with a slightly flickering foveal stimulus.

Null Adjustment Screen

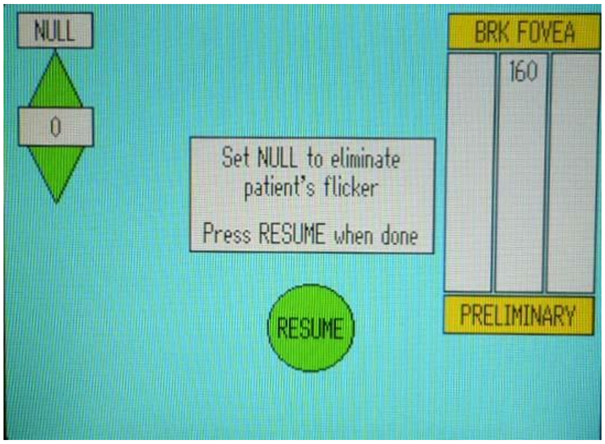

Null adjustment

The null adjustment procedure determines a good starting blue-to-green ratio for the patient. The patient, in this case, is presented with a flickering centrally-fixated target. Instruct the patient to fixate and confirm that flicker is perceived. The operator then increments the NULL adjustment by touching the green UP ARROW one step at a time while inquiring of the patient at each step if the flicker has stopped. The NULL field defaults to a beginning setting of 0. The maximum setting is 11. **ATTENTION:** *Some patients, especially older patients, may need to refocus the stimulus when switching from CFF to Bracketing due to potential effects of chromatic aberration. The CFF stimulus (green) is spectrally different from the Bracketing stimulus (blue + green). Inquire of the patient at this point if they need to refocus the stimulus.* When the patient reports that there is no flicker **STOP** adjusting and press the RESUME button on the touch screen to proceed with the bracketing phase. After touching the RESUME button reaffirm that the patient is seeing no flicker. If the patient reports any flicker, touch the UP ARROW again to null the flicker and have them proceed by pushing their response button. **NOTE:** If a patient reports no-flicker in the starting condition (Null level = 0) they most likely have very little macular pigment. Adjust the NULL level until the patient reports flicker and then return to level 0 to confirm that they do not see flicker. When you return to level 0 you will be presented with a popup field indicating that the patient has near zero MPOD. There is no need to proceed with the session since the patient is identified at this stage as having low MPOD. Touch this field to exit the session. In another special condition, if a patient is presented with all Null levels, zero through eleven, and does not report no-flicker at any level they have a MPOD level in excess of 1.0 which is well above average. When the Null level is set to eleven the operator is presented with a popup field indicating that the patient's MPOD is greater than 1.0. Touch this field to exit the session since the patient is already identified as having high MPOD. It is good practice, however, to confirm that your patient does not experience no-flicker at any null level. Reset the level to zero and run the null session again.

Foveal bracketing (1 deg. target)

Pressing the RESUME button on the touch screen initiates the foveal bracketing phase and sounds an audible triple-beep alerting the patient to proceed. The patient pushes the response button to initiate the first trial and again when flicker is perceived. This is repeated until all foveal bracketing trials are completed.

Foveal Bracketing Screen

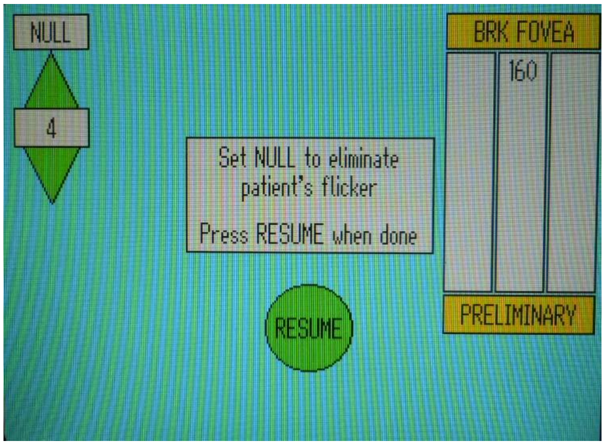

When foveal bracketing is completed the stimulus is turned off for a few seconds as the program advances to the next phase. The next phase will be either the parafoveal (peripherally-fixated) CFF phase or the optional Meso (0.5 deg) foveal phase if it has been selected for testing.

Optional Meso Evaluation

If Meso (0.5 degree target) evaluation is desired it must be manually selected for inclusion in the session prior to beginning the session. Select Meso evaluation according to the following instructions:

1) On the main screen touch the “admin” button.
2) When presented with the Password screen, enter “111” and touch “Done.”
3) You are returned to the main screen in administrative mode and a field labeled “Wakeup Config” is present on the left of the screen. Touch the “Wakeup Config” field.
4) In “Wakeup Config” touch the center field to select “Meso Evaluation.” Above the field on the right touch the green Up Arrow to set the field to “ON.”
5) Touch the “SAVE” button.
6) Touch the “MENU” button. You are returned to the main screen.
7) To exit administrative mode before starting your session, touch the “admin” button again.
8) When the Password screen is presented, enter “0” and press “Done.” You are returned to the main screen and ready to begin the session with Meso evaluation included.

When Meso (0.5 deg. target) evaluation is selected, it is always run after the standard foveal (1 deg. target) evaluation. The phases are presented as follows: Standard Foveal CFF, Standard Foveal Nulling, Standard Foveal Bracketing, Meso CFF, Meso Nulling, Meso Bracketing.

When Standard Foveal (and Meso if selected) bracketing is completed the following instruction screen is presented prompting the operator to instruct the patient on parafoveal (peripheral) fixation. The patient is presented with the parafoveal CFF stimulus, a red LED fixation point to the left of the larger (2 deg.) parafoveal target.

PAUSE

At this point have the patient sit back and relax while the operator, using the provided drawing, instructs them on the peripheral fixation task, i.e., maintain **careful fixation** on the red LED while attending to the peripherally-viewed target for the onset of flicker. Emphasize the importance of fixating the red LED and not looking directly at the target.

Instructional Break Screen

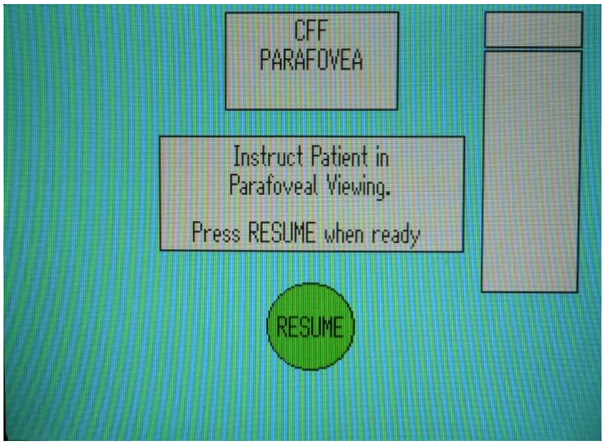

Parafoveal CFF

After the patient has been instructed on peripheral fixation, the operator presses the RESUME button on the touch screen. This initiates the parafoveal CFF phase and sounds an audible triple-beep signaling to the patient that they may proceed. Instruct the patient that when approaching the eyepiece they should immediately direct their attention to the red fixation LED to the left, avoiding direct viewing of the stimulus. This will prevent afterimages produced by viewing the stimulus and background directly. Also instruct patients to blink regularly during peripheral viewing. Once they are properly fixated the patient should press the response button to initiate the first trial and again when flicker is perceived to record their response. This is repeated for 5 trials. The program advances to the next phase, parafoveal flicker nulling.

Parafoveal flicker nulling

The operator is presented with the following screen and the patient is presented with a slightly flickering parafoveal stimulus. The null adjustment procedure determines a good starting blue-to-green ratio for the patient. The patient, in this case, is presented with a flickering peripherally-fixated target. Instruct the patient to fixate the red LED and confirm that flicker is perceived. The operator then increments the NULL adjustment by touching the green UP ARROW one step at a time while inquiring of the patient at each step if the flicker has stopped. The NULL field defaults to a beginning setting of 0. The maximum setting is 250. When the patient reports that

flicker has stopped **STOP** adjusting and press the RESUME button (triple-beep is sounded) on the touch screen to proceed with the bracketing phase. After touching the RESUME button reaffirm that the patient is seeing no flicker. If the patient reports flicker present, touch the UP ARROW again to null the flicker. Proceed with bracketing.

Parafoveal Bracketing Screen

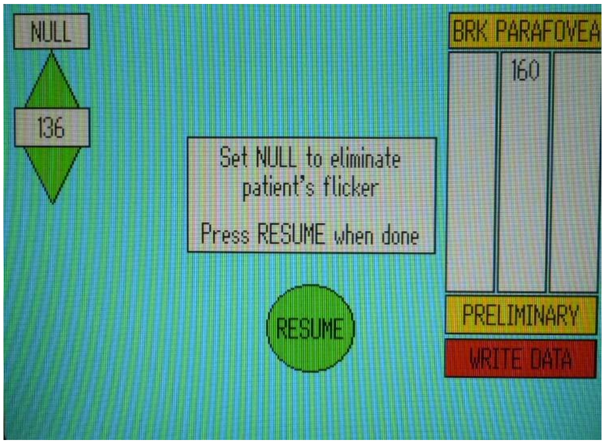

Parafoveal bracketing

Transition into parafoveal bracketing requires no new instruction or action on the part of the operator or the patient. The patient continues to fixate the red LED fixation point and initiates the first trial by pressing the response button. The patient presses the response button again at the onset of flicker. This is repeated until all parafoveal bracketing trials are completed. When all parafoveal trials are completed the stimulus is turned off and a set of two triple-beeps is sounded signaling the end of the session.

Results Screen

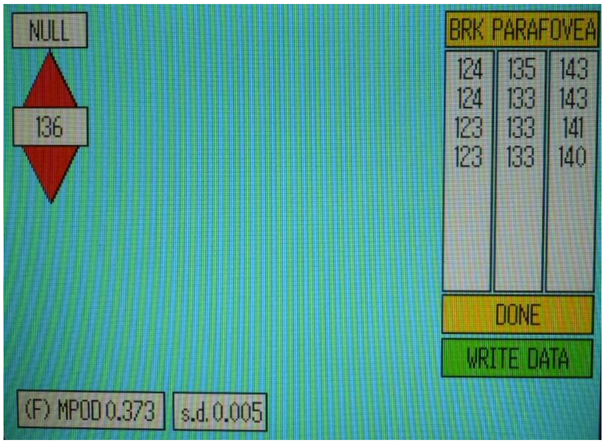

End of Session

At the end of the session the MPOD and Standard Deviation values are displayed onscreen and the WRITE DATA field is activated for recording the results to a USB flash drive. The data can be recorded manually or written to the flash drive in the USB port, or both. Pressing the WRITE DATA button on the touch screen exports the session data to the USB port and also returns the program to the start up screen. **ATTENTION:** If you remove the flash drive from the port for data transfer, before you return the flash drive to the USB port you must turn off the densitometer. Reinsert the flash drive in the USB port and then turn on the densitometer to continue collecting patient data files.

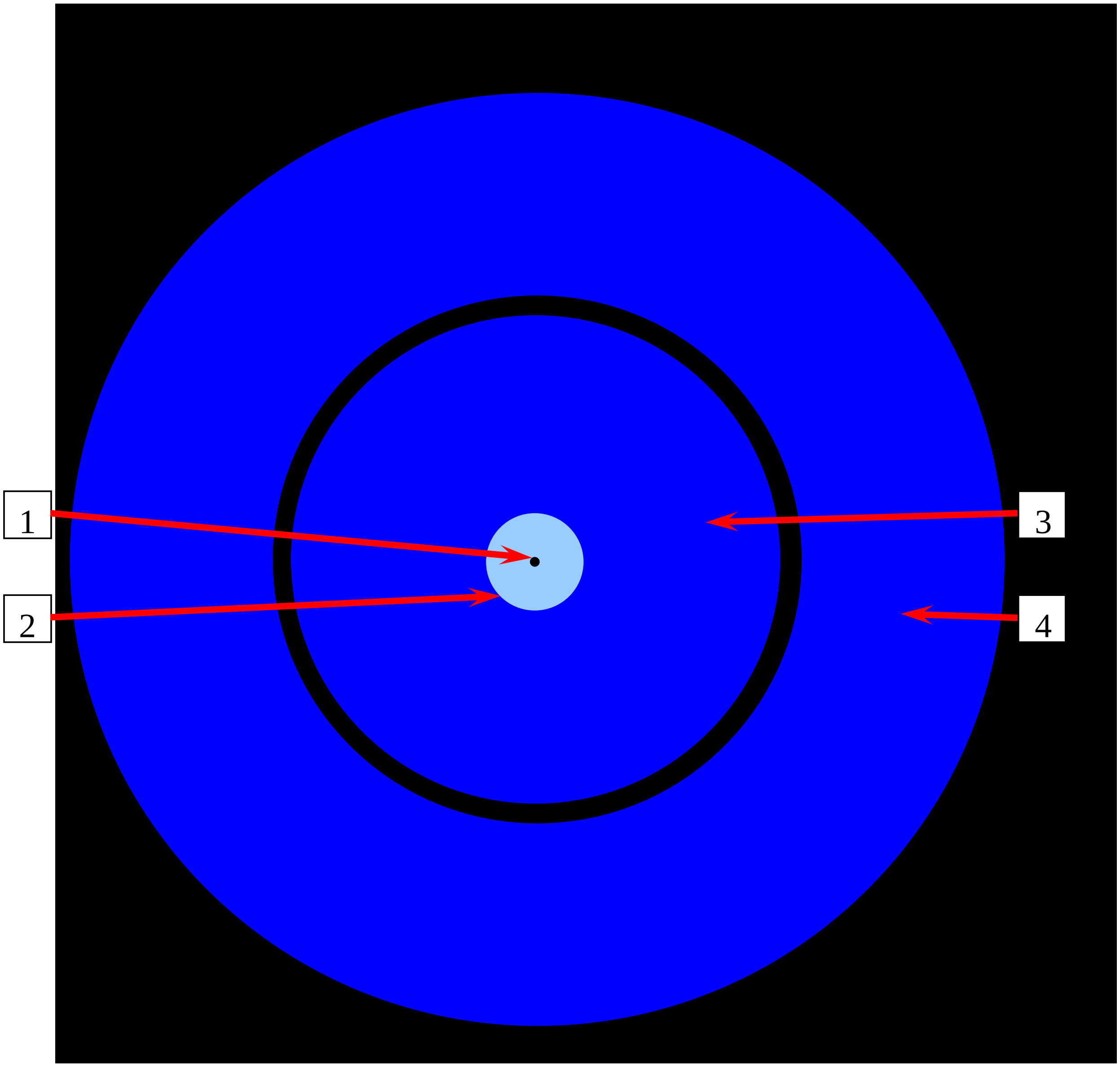

1) Fixation point.
2) Target.
3) Background field.
4) Surround field.

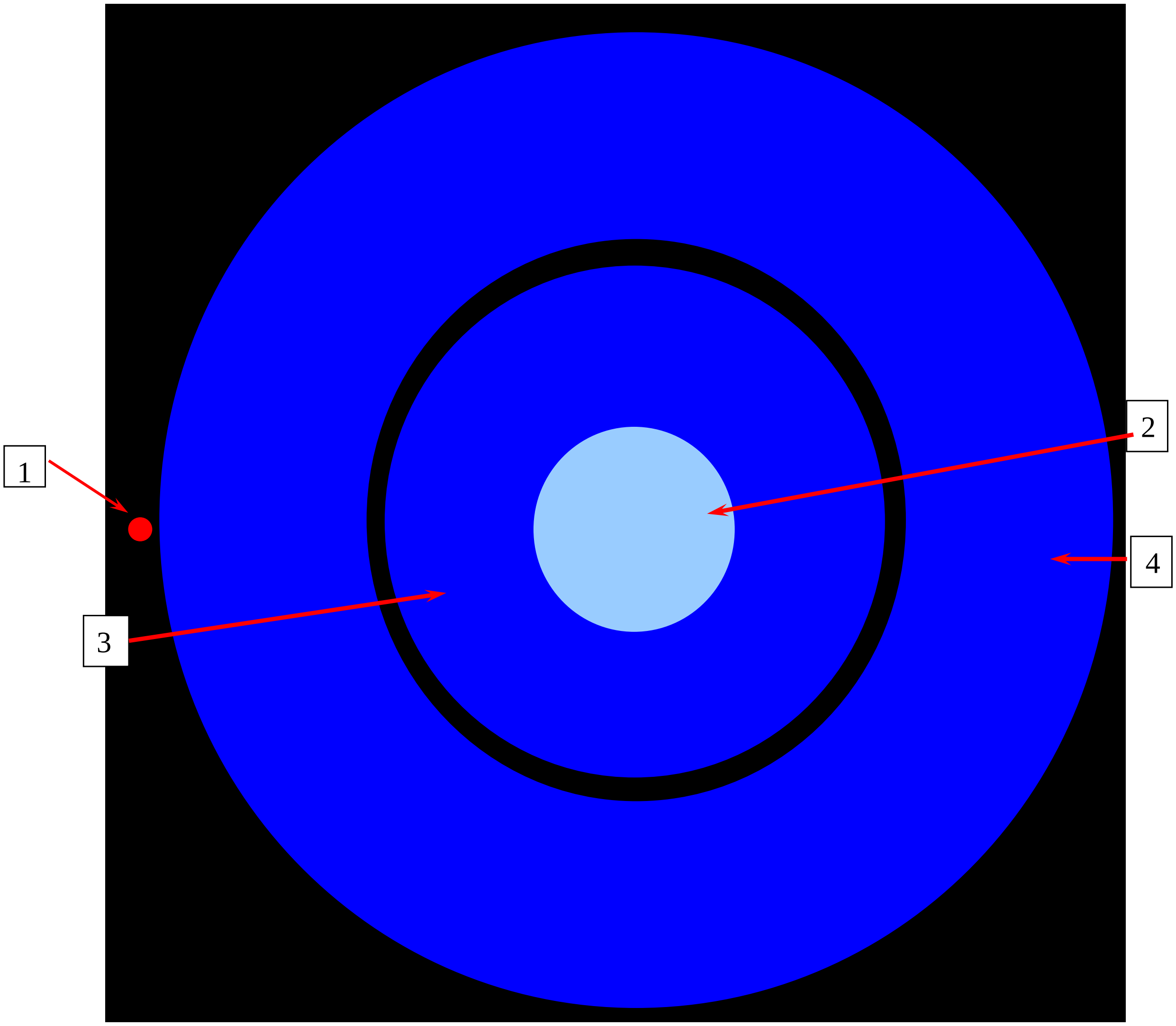

1) Fixation point (LED).
2) Target.
3) Background field.
4) Surround field.
