# Supplementary material for: Assessment of Maternal Macular Pigment Optical Density (MPOD) as a Potential Marker for Dietary Carotenoid Intake during Lactation in Humans
Source: Nutrients. 2021 Dec 31;14(1):182. doi: 10.3390/nu14010182 (PMC8746783; doi:10.3390/nu14010182)
Supplement: Supplementary file 1 [file nutrients-14-00182-s001.zip › nutrients-1498424-Supplementary Tables and Figures.pdf]

Supplemental Table S1. List of carotenoids measured from breastmilk samples.

Carotenoid	Carotenoid Value, Units
**Lutein**	13-cis-lutein, mcg/kg
	13′-cis-lutein, mcg/kg
	trans-lutein, mcg/kg
	Total lutein (13-cis-lutein + 13′-cis-lutein + trans-lutein)
**Zeaxanthin**	zeaxanthin, mcg/kg
**β-Carotene**	13-cis-β-carotene, mcg/kg
	trans-β-carotene, mcg/kg
	Total β-carotene (13-cis-β-carotene + trans-β-carotene)
**Lycopene**	trans-lycopene, mcg/kg

Supplemental Table S2. Comparison of carotenoid intake between mothers based on breastmilk sample availability

	α-Carotene		β-Carotene		β-Cryptoxanthin		Lutein-Zeaxanthin		Lycopene	
	With_BM	Without_BM	With_BM	Without_BM	With_BM	Without_BM	With_BM	Without_BM	With_BM	Without_BM
**Number of values**	38	42	38	42	38	42	38	42	38	42
**Minimum**	46.3	29.7	487.5	317.1	19.2	21.3	736.5	88.4	1053.0	339.5
**25% Percentile**	242.7	326.1	2837.0	2461.0	100.1	113.0	2154.0	1794.0	3697.0	2458.0
**Median**	471.3	539.0	4556.0	4530.0	137.8	156.9	3378.0	3675.0	4859.0	4946.0
**75% Percentile**	844.8	969.5	8780.0	9215.0	223.2	263.1	5862.0	7307.0	7328.0	7665.0
**Maximum**	2491.0	2341.0	12609.0	14176.0	490.6	554.6	12716.0	13969.0	19979.0	17983.0
**Range**	2445.0	2311.0	12122.0	13859.0	471.4	533.2	11979.0	13881.0	18926.0	17644.0
**Mean**	625.7	764.2	5549.0	5878.0	166.8	198.3	4477.0	4734.0	5715.0	5674.0
**Std. Deviation**	515.8	630.9	3291.0	4009.0	106.7	124.2	3228.0	3541.0	3562.0	4067.0
**Std. Error of Mean**	83.7	97.4	533.9	618.6	17.3	19.2	523.6	546.5	577.8	627.6

Supplemental Table S3. Unadjusted and adjusted linear model of the association between MPOD and dietary carotenoids.

Carotenoid	MPOD (No Covariates)				MPOD (Covariates: Maternal Age, BMI, DaysPP)			
	β (slope)	R^2	*p*-Value	q-Value	β (slope)	R^2	*p*-Value	q-Value
α-Carotene	1021.24	0.10	0.00	0.04 *	902.87	0.17	0.01	0.05 *
β-Carotene	2170.92	0.01	0.34	0.84	1240.87	0.04	0.60	0.84
β-Cryptoxanthin	17.27	0.00	0.81	0.84	14.98	0.04	0.84	0.84
Lutein-Zeaxanthin	2088.21	0.01	0.32	0.84	907.04	0.08	0.67	0.84
Lycopene	–548.17	0.00	0.82	0.84	–706.57	0.03	0.77	0.84

MPOD, Macular pigment optical density. DaysPP, days postpartum. * For these variables the total concentration of the carotenoid was included as covariates.

Supplemental Table S4. Lack of correlation between MPOD and carotenoid-rich foods.

Carotenoid-rich foods (FFQ)	MPOD	
	r	*p*-Value
Total vegetables, cups	0.09	0.44
Vegetables dark green leafy, cups	0.05	0.67
Vegetables deep yellow orange, cups	0.15	0.17
Solid fruit (not juice), cups	0.08	0.50

MPOD, Macular pigment optical density

Supplemental Table S5. Correlation of Maternal Carotenoid Intake with Breastmilk Contents

No Covariates															
Carotenoid	α-Carotene			β-Carotene			β-Cryptoxanthin			Lutein-Zeaxanthin			Lycopene		
	r	p-value	q-value	r	p-value	q-value	r	p-value	q-value	r	p-value	q-value	r	p-value	q-value
13'-cis-lutein	0.015	0.931	0.975	0.218	0.195	0.805	0.215	0.201	0.805	0.260	0.121	0.805	-0.066	0.699	0.966
13-cis-lutein	-0.103	0.558	0.890	0.058	0.741	0.966	0.184	0.289	0.805	0.188	0.279	0.805	0.113	0.520	0.890
trans-lutein	-0.149	0.379	0.825	0.169	0.318	0.805	0.195	0.247	0.805	0.237	0.158	0.805	0.076	0.656	0.966
Zeaxanthin	-0.093	0.585	0.890	0.222	0.187	0.805	0.189	0.262	0.805	0.260	0.120	0.805	-0.018	0.915	0.975
13-cis-β-carotene	0.096	0.571	0.890	0.175	0.302	0.805	0.200	0.236	0.805	0.110	0.517	0.890	-0.074	0.664	0.966
trans-β-carotene	-0.008	0.961	0.975	0.011	0.950	0.975	0.238	0.156	0.805	0.002	0.993	0.993	-0.039	0.820	0.966
trans-lycopene	-0.214	0.204	0.805	-0.109	0.519	0.890	0.111	0.513	0.890	-0.019	0.909	0.975	0.012	0.944	0.975

Maternal age, BMI and DaysPP as Covariates															
Carotenoid	α-Carotene			β-Carotene			β-Cryptoxanthin			Lutein-Zeaxanthin			Lycopene		
	r	p-value	q-value	r	p-value	q-value	r	p-value	q-value	r	p-value	q-value	r	p-value	q-value
13'-cis-lutein	-0.019	0.918	0.975	0.189	0.291	0.805	0.282	0.111	0.805	0.246	0.168	0.805	-0.100	0.578	0.890
13-cis-lutein	-0.150	0.420	0.865	0.042	0.821	0.966	0.212	0.252	0.805	0.202	0.276	0.805	0.110	0.556	0.890
trans-lutein	-0.168	0.351	0.819	0.105	0.561	0.890	0.161	0.371	0.825	0.178	0.322	0.805	-0.011	0.953	0.975
Zeaxanthin	-0.100	0.581	0.890	0.186	0.299	0.805	0.261	0.142	0.805	0.243	0.174	0.805	-0.062	0.731	0.966
13-cis-β-carotene	0.068	0.707	0.966	0.155	0.389	0.825	0.398	0.022	0.770	0.101	0.577	0.890	-0.068	0.709	0.966
trans-β-carotene	-0.030	0.870	0.975	-0.044	0.807	0.966	0.420	0.015	0.770	-0.053	0.770	0.966	-0.046	0.797	0.966
trans-lycopene	-0.264	0.138	0.805	-0.174	0.334	0.806	0.328	0.062	0.805	-0.047	0.795	0.966	0.039	0.828	0.966

DaysPP, days postpartum.

Supplemental Table S6. Unadjusted and adjusted linear model of the association between MPOD and breastmilk carotenoids. Significant association values are highlighted with green outline. * For these variables the total concentration of the carotenoid was included as covariates.

Carotenoid	MPOD (no covariates)				MPOD (covariates: Maternal age, BMI, DaysPP)			
	β (slope)	R^2	*p*-Value	q-Value	β (slope)	R^2	*p*-Value	q-Value
13'-cis-lutein *	1.60	0.56	0.14	0.31	1.56	0.56	0.20	0.31
13-cis-lutein *	4.60	0.82	0.01	0.04	4.66	0.83	0.01	0.05
trans-lutein *	–6.19	0.96	0.00	0.04	–6.17	0.96	0.01	0.04
Zeaxanthin	1.97	0.00	0.70	0.77	2.56	0.04	0.64	0.77
13-cis-β-carotene *	4.70	0.62	0.17	0.31	4.64	0.62	0.22	0.31
trans-β-carotene *	–4.70	0.98	0.17	0.31	–4.64	0.98	0.22	0.31
trans-lycopene	–0.33	0.00	0.77	0.77	–0.33	0.15	0.77	0.77

MPOD, Macular pigment optical density. DaysPP, days postpartum.

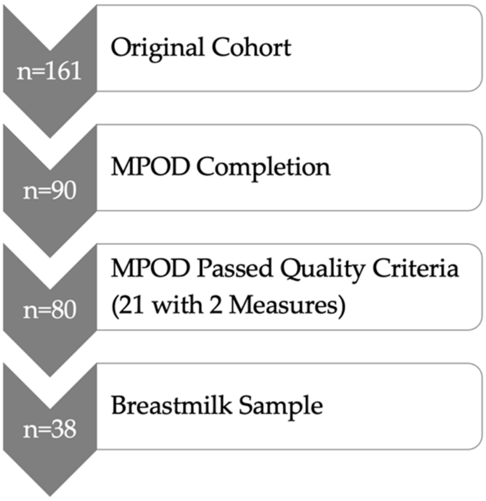

Supplemental Figure S1. Participant and data availability. MPOD, Macular pigment optical density.

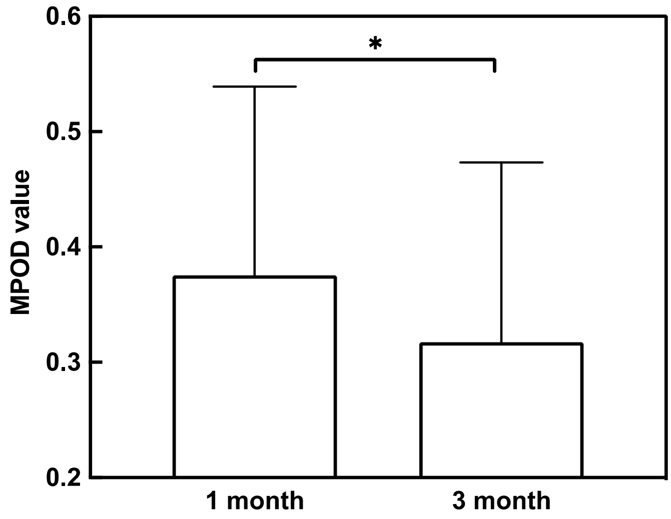

Supplemental Figure S2. Serial MPOD measures. MPOD, Macular pigment optical density

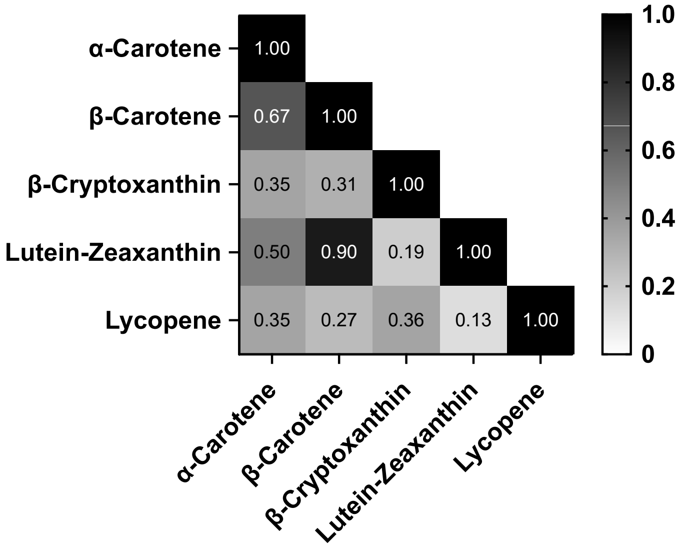

Supplemental Figure S3. Correlation matrix of dietary carotenoids (Correlation coefficients are displayed in each box).
